# Postponing Pregnancy Through Oocyte Cryopreservation for Social Reasons: Considerations Regarding Clinical Practice and the Socio-Psychological and Bioethical Issues Involved

**DOI:** 10.3390/medicina54050076

**Published:** 2018-10-25

**Authors:** Mara Simopoulou, Konstantinos Sfakianoudis, Panagiotis Bakas, Polina Giannelou, Christina Papapetrou, Theodoros Kalampokas, Anna Rapani, Ekaterini Chatzaki, Maria Lambropoulou, Chrysoula Lourida, Efthymios Deligeoroglou, Konstantinos Pantos, Michael Koutsilieris

**Affiliations:** 1 Department of Physiology, Medical School, National and Kapodistrian University of Athens, 75, Mikras Asias, 11527 Athens, Greece; lina.giannelou@gmail.com (P.G.); rapanianna@gmail.com (A.R.); mkoutsil@med.uoa.gr (M.K.); 2 Assisted Conception Unit, 2nd Department of Obstetrics and Gynecology, Aretaieion Hospital, Medical School, National and Kapodistrian University of Athens, 76, Vasilisis Sofias Avenue, 11528 Athens, Greece; p_bakas@yahoo.com (P.B.); christina.papapetrou@gmail.com (C.P.); kalamp@yahoo.com (T.K.); chrisalouri@hotmail.com (C.L.); edeligeo@aretaieio.uoa.gr (E.D.); 3 Centre for Human Reproduction, Genesis Athens Clinic, 14–16, Papanikoli, 15232 Athens, Greece; sfakianosc@yahoo.gr (K.S.); info@pantos.gr (K.P.); 4 Laboratory of Histology-Embryology, Medical School, Democritus University of Thrace, 68100 Alexandroupolis, Greece; achatzak@med.duth.gr; 5 Laboratory of Pharmacology, Medical School, Democritus University of Thrace, 68100 Alexandroupolis, Greece; mlambrop@med.duth.gr

**Keywords:** assisted reproduction, oocyte vitrification, social reasons, extending fertility, reproductive psychology, bioethics

## Abstract

Oocyte freezing for ‘social reasons’ refers to women of reproductive age who are aiming to prolong, protect and secure their fertility. The term emerged to describe application of the highly promising technique, namely vitrification on oocytes retrieved through controlled ovarian stimulation (COS) from women intending to preserve their fertility for social reasons. These women opt to cryopreserve their oocytes at a point in their life when they need to postpone childbearing on the grounds of so called ‘social’ reasons. These reasons may include a highly driven career, absence of an adequate partner, financial instability, or personal reasons that make them feel unprepared for motherhood. This is a sensitive and multifaceted issue that entails medical, bioethical and socio-psychological components. The latest trend and the apparent increase noted on oocyte freezing for ‘social reasons’ has prompted our team of fertility specialists, embryologists, obstetricians, gynecologists and psychologists to proceed with a thorough, critical and all-inclusive comprehensive analysis. The wide range of findings of this analysis involve concerns of embryology and epigenetics that shape decisions made in the IVF laboratory, issues regarding obstetric and perinatal concerns on the pregnancy concluding from these oocytes and the respective delivery management and neonatal data, to the social and bioethical impact of this trend’s application. This literature review refers to matters rising from the moment the ‘idea’ of this option is ‘birthed’ in a woman’s thoughts, to proceeding and executing it clinically, up until the point of the pediatric follow up of the children born. We aim to shed light to the controversial issue of oocyte freezing, while objectively exhibit all aspects regarding this complex matter, as well as to respectfully approach how could the prospect of our future expectations be shaped from the impact of its application.

## 1. Background

### 1.1. From Cryobiology to Social Freezing

Cryobiology enables the preservation of tissues and other biological material through a procedure of freezing and thawing, whilst maintaining their vitality [1]. This article is focused on oocyte cryopreservation. The first live birth from cryopreserved oocytes was recorded in 1986, employing slow freezing [2]. A new cryopreservation method called ‘vitrification’ followed, leading to the first live birth from frozen oocytes in 1999 [3]. Vitrification is described as a technique that enables cells to be cooled at −196 °C avoiding the risk of crystallization by implementation of cryoprotectant solutions [4]. Use of vitrification has been associated with improved success rates of in vitro fertilization (IVF). The success rates employing vitrified oocytes are reported to be similar to those of employing fresh oocytes [5,6,7,8,9]. Oocyte cryopreservation mainly targeted fertility preservation for women diagnosed with cancer prior to gonadotoxic therapy. During the last decades, however, oocyte vitrification has become more popular between women of reproductive age who wish to cryopreserve their oocytes for non-medical reasons. This new ‘trend’ is introduced as ‘social egg freezing’ and allows women to postpone the time of childbearing to a more ‘convenient’ or ‘appropriate’ time in the future. This in turn may exclude age-related fertility loss and the concerns on premature ovarian failure (POF) from the equation. Postponing having a child until over the age of 35 years eventually results to dramatically decreased pregnancy rates, which become even more pronounced if motherhood is postponed until the age of 40 [10].

### 1.2. Successful Application of Vitrification and the Concerns Raised

It is primarily the age of the woman at the time of oocyte retrieval, and hence the oocyte age that influences the pregnancy outcome [11] and secondarily the age at the actual time of use of the oocytes. The increased emergence of programs and services that address age-related subfertility demonstrate remarkably rising pregnancy success rates with the use of frozen oocytes [12]. The aforementioned success rates have encouraged women to consider social freezing as an option in an effort to avoid the possibility of remaining childless. Although this option appears to be ideal for the working woman in the 21st century, it raises various concerns. There is a debate on whether to offer social oocyte freezing or not in everyday medical practice as it is considered more of a “choice” and not “the only alternative” which is the case for cancer patients [13]. Since 2012 and 2013, ESHRE and ASRM respectively no longer characterize social egg freezing as experimental and the data on outcomes can now be considered fairly satisfactory [14,15]. In 2017, a cross-sectional survey study of 663 women aged between 18 and 44 years reported a higher rate of awareness of ovarian reserve and oocyte freezing for non-medical reasons than in previous studies and a higher tolerance to use modern technology in order to avoid unintended childlessness [16].

**Figure 1:**
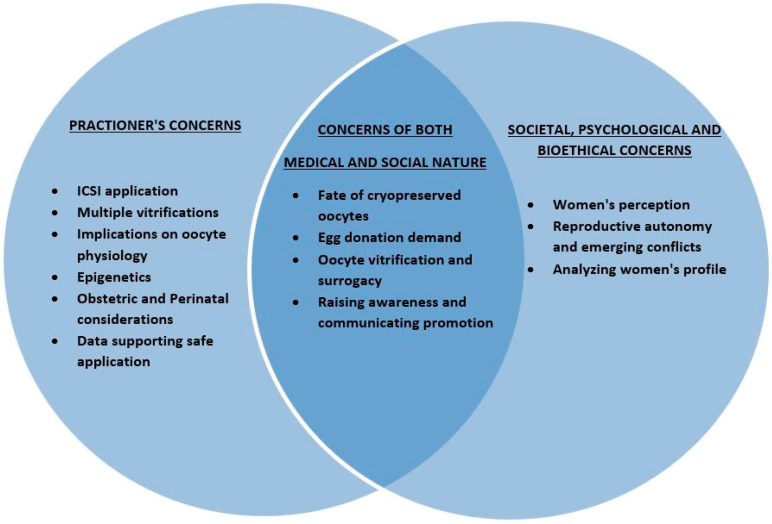
Oocyte vitrification for social reasons and perspectives examined.

**Figure 2:**
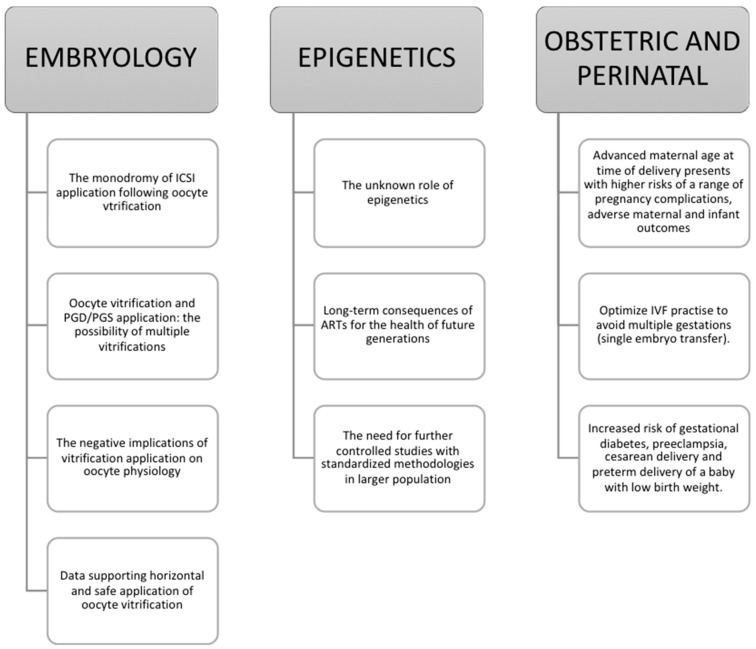
How oocyte cryopreservation for social reasons can affect matters of embryology practice, epigenetic issues and obstetric and perinatal concerns.

**Figure 3:**
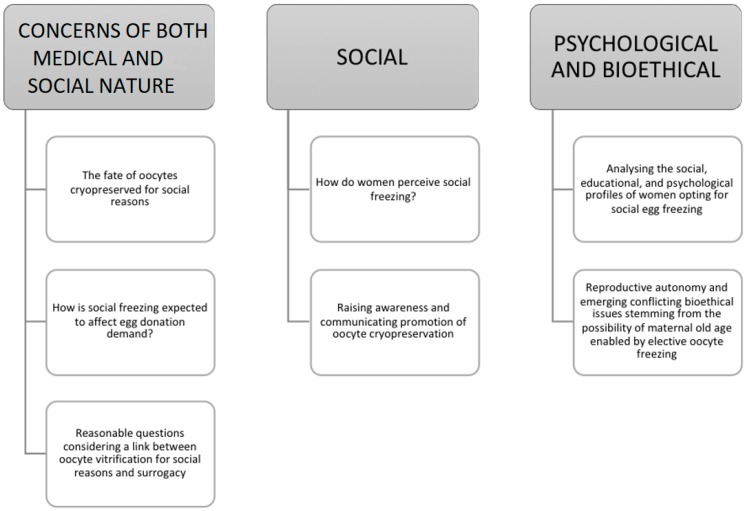
Overview of the implications of oocyte cryopreservation on issues of both medical and social nature and psychological and bioethical viewpoints.

There are decisions to be made, country legislations to change, legal issues to be approached, and further research programs on social egg freezing effectiveness prior to a true horizontal application. Social egg freezing may make us reconsider the life choices timeframe as we know it. It may challenge and influence structures such as society, family planning, human relationships, parenting, tradition, ethical issues, as well as financial aspects. Social egg freezing is here to stay; the question is “under what circumstances?”. Hereby, we express reasonable questions on how to ascertain improved and effective practice. Oocyte freezing from the practitioner’s perspective entails an array of serious concerns including the mandatory application of ICSI, the possibility of multiple vitrifications and the risks vitrification comprises on oocyte physiology, obstetric and perinatal complications arising from maternity at advanced age, as well as the culprit of all unexplained failures: epigenetics. One could easily recognize that the above constitute serious concerns. However, despite the research providing data reinforcing the true beneficial nature of oocyte freezing, society remains a challenging component to consider. We highlight the implications of this phenomenon and the profound importance of promoting and raising society’s awareness regarding oocyte freezing. Another important issue refers to the use of ‘social egg freezing’ as a term and its ‘correctness’. An interesting contribution by Stoop and colleagues suggested adoption of the term ‘oocyte banking for anticipated gamete exhaustion (AGE)’ serving better the description of the trend, describing the driver behind it as a preventive intervention that is neither social nor medical [17]. Extensive counseling is required for women contemplating cryopreserving their oocytes for social reasons regarding their options, alternatives, limitations and realities. The perspectives of the implications of oocyte cryopreservation for social reasons analyzed in this study are outlined in Figure 1, Figure 2 and Figure 3.

### 1.3. Methodology

A computerized search was performed employing PubMed regarding the current status of oocyte cryopreservation considering its history, clinical indications and the efficacy of this technique that entails medical, scientific, bioethical and psychosocial issues. The exact basic keywords and combinations employed for this search are listed herein, “oocyte freezing”, “social freezing”, “oocyte freezing for social reasons”, “oocyte cryopreservation”, “oocyte vitrification”, as well as combinations including the aspects examined with respect to social freezing such as “bioethics and oocyte freezing” and “psychosocial implications of oocyte cryopreservation”. The search was performed on 23 June 2018 and provided 192 articles. The search strategy is provided in Supplementary Table S1. Exhausting literature research led to inclusion and exclusion following screening and evaluation of original studies and reviews based on the titles, abstracts and relevance to this topic investigated. Articles in English was a basic criterion upon consideration, while publication date limitation was not accounted. Another inclusion criterion concerned publications focusing on association of “social oocyte freezing” and one or more topic namely: “embryology”, “epigenetics”, “obstetrics and perinatal”, “psychology” and “bioethics”. Exclusion criteria referred to articles on vitrification as a fertility preservation technique for cancer patients, patients with a disorder threatening fertility status, and premature ovarian failure (POF), articles that were not published in peer-reviewed journals and were based only on non-human studies. Citation mining was performed, which refers to the process of investigating the reference list of every individual reference described in the manuscript in order to display the full impact of research regarding our topic of interest. Finally, a list of publications that was meticulously evaluated and critically analyzed instituted our reference background.

## 2. Practitioner’s Concerns on the Paradox of IVF in the Service of Fertile Women

Assisted reproductive technology (ART)’s target group is people with difficulties in conceiving naturally. Male factor, female factor, both, or unexplained infertility are common terms for IVF specialists who deal with such couples. Women who are tempted by the idea of oocyte vitrification tend to be healthy career women with no proven infertility and presumably highly capable of conceiving naturally at a future moment of their choosing [18]. This future point may include standards that must have been fulfilled up to this time such as, a husband, an ideal job, or professional promotion. One of the concerns raised could be that the initial focus of ART diverges from its primary fundamental application. IVF in the service of preserving fertility via oocyte vitrification will become a required step in the process employed on possibly fertile women. We are gradually entering a new era due to social egg freezing. Assisted reproduction services are used by couples or single women who are not facing infertility. Further studies on the use of this tool on a special non-infertile population are required.

### 2.1. Monodromy of ICSI Application Following Oocyte Vitrification

The methodology of vitrification imposes specific treatment of the frozen oocytes for pregnancy to be achieved. Prior to vitrification the oocytes’ maturity has to be assessed. Therefore, cumulus cells need to be removed, this in turn renders the oocyte unsuitable for the traditional technique for fertilization, but instead dictates the use of the more invasive technique namely intra cytoplasmic sperm injection (ICSI). In addition to the above, the hardening of the zona pellucida that has been documented and reported post thawing [19,20] renders ICSI the only available manipulation for these oocytes. The challenging nature of ICSI application and the several extra manipulations possibly entailed-involving both the sperm and the oocyte- are thoroughly examined and discussed in our previous publications [21,22]. Thus, in the case of ‘social freezing’, we are employing the scope of in vitro fertilization techniques for couples or single women that are not necessarily infertile. Particularly, ICSI will be employed irrespectively of male factor etiology, which should be the main prerequisite for ICSI application. Amplification of such a practice will create a paradox scene dictating a different clinical practice. Future follow-up and long-term effects are to be assessed.

### 2.2. Oocyte Vitrification and PGD/PGS Application: The Possibility of Multiple Vitrifications

Preimplantation genetic diagnosis (PGD) and preimplantation genetic screening (PGS) are solid techniques offering information on the genetic constitution of the embryo for diagnostic and/or screening purposes. With ICSI procedure being an already invasive technique lacking a universally applied protocol [21,22] if we add preimplantation genetic diagnosis (PGD) procedure in the treatment, we add another level of complexity. The oocytes retrieved will be vitrified and thawed once. Following ICSI, they may be vitrified subsequently for a second time, as surplus embryos for future use. Further to that, in a possible scenario of PGD or PGS application these embryos may be vitrified for a third time, following blastocyst biopsy in the scope of the latest trends and most effective techniques involved on PGD/PGS protocols. Vitrification protocols have impressive results both in post-thaw survival and in clinical pregnancy rates [23]; however, we need to examine the effectiveness of vitrification protocols under the light of the possible epigenetic impact on double and triple frozen embryos. There is a clear need for further randomized controlled studies with comparable values and a high number of cases presented in comparison to control groups [24].

### 2.3. Negative Implications of Vitrification on Oocyte Physiology

Vitrification is enabling options and offering flexibility in assisted reproduction and it is an excellent tool in the IVF laboratory [23]. However, despite all the reassuring data on the safe application of oocyte vitrification to the point where results are comparable to the use of fresh oocytes [25] there is always the concern regarding the safety of the process. This mainly involves the chemical and physical damage along with the toxicity of cryoprotective agents (CPA). This damage can be linked to the spindle apparatus and the arrangement of the chromosomes [26,27]. The toxicity of cryoprotectant substances is a complex issue, given the fact that not only concentration and volume, but also temperature and time of exposure are crucial for the resulting toxicity the cells are exposed to [28]. Once these oocytes are cryopreserved they are subjected to specific structural and biological modifications. The oocyte is susceptible to cryodamage due to its large surface/volume ratio and changes in the plasma membrane permeability also occur [29]. Furthermore, intracellular functions, meiotic spindle and microtubular structures are also affected [30,31]. Points of interest regarding the negative aspects associated with vitrification application are discussed in length in our previous publication [23].

### 2.4. Epigenetics

Despite the widespread employment of ART, the exact mechanisms leading to epigenetic disorders and aberrant gene expression are not yet fully understood, not only in humans but also in the animal models [32]. In 2014, a retrospective study measured DNA methylation in four different genes from 69 children following use of IVF and ICSI and compared them to 86 children born from natural conception. The result of this retrospective cohort study points the undoubtful role of epigenetics in future generations. The study clearly indicated the urgent need for a deeper understanding of the role of epigenetics in fertility and the possible long-term consequences of ART for the health of future generations [33]. Further to that, in the first systematic review and meta-analysis in this area of interest in 2014, Bhattacharya et al. suggested that there is an increased level of imprinting disorders in children conceived employing IVF and ICSI but there is no evidence of generalized changes in DNA methylation of selected genes [34]. Considering the unknown role of epigenetics in the future generations and the horizontal application of ICSI following oocyte vitrification protocols, there is a clear need for more controlled studies with standardized methodologies in larger population.

### 2.5. Obstetric and Perinatal Considerations

Given the data enabled by the promise of oocyte vitrification for social reasons, is it safe to extrapolate that such a practice may be associated with a planned pregnancy and childbearing at an older age? When we consider the management of future planned pregnancies, is it safe to assume that the expectant maternal age at time of delivery holds high possibility to be advanced and/or very advanced? This is coupled by the fact that varying legislation in several countries allows the age limit for the provision of IVF services to be close to 50 years old, or in some countries no limitation exists [35,36]. According to the ASRM Ethics Committee statement issued in 2013, physicians should obtain a complete medical evaluation prior to embarking on an embryo transfer procedure to any woman over the age of 50. Embryo transfer should be strongly discouraged or denied to any woman over the age of 50 with underlying issues that could increase further obstetrical risks, and discouraged in women over age 55 without such issues.

Advanced maternal age at time of delivery presents with higher risks of a range of pregnancy complications and there are various reports associating it with a higher risk of adverse maternal and infant outcomes [37,38,39]. It is therefore important to ponder on the obstetric and perinatal risks that may be related to such pregnancies occurring at an older age enabled through oocyte freezing for social reasons. Before we enter the obstetric and perinatal data, it is critical to refer to the IVF practice leading to these pregnancies. Given the advanced maternal age and all the obstetric and neonatal challenges stemming from it, it is vital that all efforts are made to confine such risks. This can be achieved by reducing the number of embryos transferred, with single embryo transfer strongly recommended in this group of patients in order to avoid multiple gestations [40].

Mode of delivery in advanced maternal age women is linked with elective cesarean. The study by Faisal-Cury et al., reported that 28.4% of deliveries in public hospitals were performed by cesarean section. The bivariate analysis supported that cesarean section was associated with higher family income per capita, higher education, pregnancy planning, white skin color, having a partner and advanced maternal age [41].

Interestingly, in 2016 a population-based retrospective cohort study used the United States’ Health Care Cost and Utilization Project’s Nationwide Inpatient Sample to evaluate maternal outcomes in women with advanced maternal age delivering from 2003 to 2012. The results indicated that cesarean delivery on maternal request in healthy women of advanced maternal age is strongly linked to high risk of both in-hospital death, as well as severe morbidity during and following childbirth [42]. Physicians caring for and managing older women contemplating on a planned primary cesarean delivery, should be made aware of the potential risks involved so that they can proceed with an informed decision [42].

With respect to the obstetric complications involving the fetus and the mother, women who become pregnant at an advanced age face an increased risk of developing gestational diabetes, preeclampsia, cesarean delivery and preterm delivery of a baby with low birth weight. Even though the overall health status of the woman dictates the end result of such high-risk situations, these widely varied risks increase with advanced maternal age [43]. The population-based cohort study using the UK Obstetric Surveillance System (UKOSS) by Fitzpatrick et al., 2016, suggests that women giving birth at a very advanced maternal age present with a higher risk of having a range of pregnancy complications. The complications may include gestational hypertensive disorders, gestational diabetes, postpartum hemorrhage, caesarean delivery, iatrogenic and spontaneous preterm delivery and ITU admission. A prospective study by Zapatas-Masia et al., 2016 showed that maternal age ≥40 years was associated with poorer obstetric and perinatal outcomes and increased risks with higher frequency of cesarean section, intrauterine growth retardation, premature delivery and fetal macrosomia [44]. These findings should be considered when counselling and managing women of very advanced maternal age [45]. We may find in the future that extended practice of oocyte cryopreservation for social reasons may be associated with all the above-mentioned complications. In most cases however, the reported complications will not be severe enough to compromise the long-term health of a woman and her ability to care for a child. Age-appropriate health screenings and a thorough medical examination should be encouraged before proceeding [15].

A review on the outcome of children born following vitrification of oocytes does not describe any increased rates for chromosomal abnormalities or congenital malformations [46]. In 2008, a published analysis of 165 pregnancies and 200 infants conceived through oocyte vitrification cycles compared to outcomes from fresh IVF cycles and spontaneous conceptions reported that the mean birth weight and the rate of congenital anomalies did not differ between these groups [47]. In 2009, a wide-ranging review of the literature showed that over 900 babies had been born from oocyte vitrification cycles and a rate of 1.3% were noted to have birth anomalies. This evidence proves that there is no difference compared to congenital anomalies appearing in naturally conceived infants [47]. Another study of 1027 babies born from 804 pregnancies using vitrified oocytes compared to 1224 babies from 996 pregnancies from fresh IVF cycles including singleton and multiple pregnancies as well as own and donated oocytes suggested that the technique does not increase obstetric and perinatal outcomes [48,49]. However, further studies with larger samples and long term follow up findings of these children are required.

The rationale behind advanced maternal age pregnancies employing oocytes either through an egg donation program or using own oocytes cryopreserved for social reasons is that the actual embryo corresponds to a younger age and therefore the detrimental aspects of an advanced age pregnancy may not apply. However, the study by Tarin et al. (2016), supports that most of the effects on offspring of intrauterine exposure to maternal age-related obstetric complications may be induced by epigenetic DNA reprogramming during critical periods of embryo or fetal development [50]. Can epigenetic DNA reprogramming set off by an advanced maternal age intrauterine exposure overthrow a younger and healthier embryo? Advanced maternal age alone could be a marker for other factors negatively affecting offspring such as age-related changes in hormonal levels during pregnancy which could increase cancer risk during pregnancy [51]. It is therefore critical to inform women embarking on a fertility preservation program not only about their chances of pregnancy and the percentage of live births, but also about the risks to themselves and their prospective offspring related to delaying motherhood [50].

### 2.6. Data Supporting Horizontal and Safe Application of Oocyte Vitrification

Contrary to the above described limitations, in a cohort multi-center study, Rienzi et al. 2010 reported that oocyte vitrification is an efficient and reliable approach. In the same study, in 486 cycles for 450 couples, 84.7% of the oocytes survived cryopreservation. Moreover, a large study including 2182 oocytes subjected to ICSI presented with 75.2% rate of fertilization and 48.1% rate of development to top-quality embryos ending up with 26.3% deliveries per cycle and 29.4% deliveries per transfer [25]. With oocyte survival rates reaching 84%, it has been suggested that IVF outcomes from both fresh and vitrified oocytes could achieve similar outcomes [52,53,54]. In 2011, Cobo and Diaz published a meta-analysis of randomized controlled trials assessing the efficiency of oocyte vitrification comparing IVF cycles with fresh, slow frozen, and vitrified oocytes in terms of oocyte survival, fertilization, embryo development and pregnancy rates. They concluded that there was not any significant difference between the studying groups [55]. The last decade more and more IVF clinics embrace vitrification protocols as the standard technique to cryopreserve oocytes, whereas in 2013 the National Institute of Health and Care Excellence (NICE) published an updated clinical guideline stating “in cryopreservation of oocytes and embryos, use vitrification instead of controlled-rate freezing if the necessary equipment and expertise is available” [56].

In the following section, we report on the several points raised regarding the implications of oocyte vitrification for social reasons on the ART set-up. These issues involve an extension to societal and bioethical aspects encompassing challenges that concern both sides.

## 3. Concerns of Both Medical and Social Nature

### 3.1. Fate of Oocytes Cryopreserved for Social Reasons

There is a limited time frame that a couple or a single person can keep cryopreserved samples in an IVF cryobank. Following this consented period, which is subjected to specific legislation, the samples can be employed in an IVF cycle aiming to secure a pregnancy, perished/destroyed, donated to other couples, or donated to research. This is a choice that sometimes is made alongside the cryopreservation procedure. Research shows that 50% of women who postpone a pregnancy until after their thirties seem to conceive in the six years that follow [57]. With oocyte vitrification for social reasons in mind, there is a possibility that a considerable number of cryopreserved oocytes will never be used. What will happen to those oocytes? In the case of social egg freezing, if a woman no longer has use for her surplus cryopreserved oocytes—because, for instance, she has achieved the number of desirable healthy pregnancies—then the unused oocytes may be discarded or donated to other couples or donated to research [58]. This treatment for age-related infertility is still new. Consequently, there is not sufficient information about the outcomes of elective oocyte cryopreservation because there is limited data about women returning to use them [14,17].

Hodes-Wertz and colleagues (2013) showed that only 6% of women had used their oocytes during the six-year timeframe of cryopreservation. In a more recent study, Baldwin and colleagues reported that about 88% of the women who underwent oocyte cryopreservation for social reasons stated that they would donate ‘spare’ oocytes to medical research programs or to other infertile couples if they never required them [59].

According to French law, there is a suggestion that oocyte donors to keep a number of oocytes for themselves, just in case of future use. Another trend is the ‘freeze and share’ approach that some clinics offer. Women who cryopreserve their oocytes will have a discount or even be offered the services that the clinic provides free of charge, if they donate a number of their frozen oocytes [13].

The fate of the oocytes depends largely on each country’s legislation, the Code of Practice and the bioethics regarding the issue of donation. Even more so it depends on the definition that each country gives to ‘donation’ and what constitutes a donor. In China, for example, the law presents with restriction in regard to who will be considered as a donor. A woman in order to become an oocyte donor needs to have a reproductive history of ART procedures (IVF or ICSI) in which a number of more than 20 oocytes were collected and a number of oocytes (at least 15) were stored for future personal use [60].

To conclude, the novelty of this phenomenon has left us with lack of adequate information about this new trend which is changing the ART field and the routine of embryology laboratories. With respect to the fate of these oocytes outside the original plan of them being employed to secure a pregnancy, the future could present us with opportunities for new donation and research programs.

### 3.2. How Social Freezing Is Expected to Affect Egg Donation Demand

The advent of elective oocyte freezing enables ‘biological equity’, entailing the right to being able to have children and more specifically the right of becoming the biological parent of one’s own children [61]. It could be hypothesized that the rapid increase of the use of oocyte cryopreservation services reflects a form of realization and safeguarding of ‘biological parenthood’. The hypothesis stands that such a practice may limit alternatives such as adoption and the use of donor gametes. The choices and alternatives to managing infertility are presented by reproductive medicine and its practitioners. Bayles (1984) has extensively examined the cultural and biological importance attached to genetic relatedness regarding people’s prioritization of choices when faced with issues of subfertility.

There is a notion that oocyte freezing will lead to a decline in egg donation’s public demand. Most of the women who cryopreserve their eggs will plan on embarking on an IVF cycle and perhaps consecutive IVF cycles employing their frozen eggs before they investigate another reproductive option. The question raised is whether egg donation will become less popular. Nonetheless, oocyte freezing fails to guarantee that a woman will become pregnant with a 100% certainty. Studies have revealed that the possibility for a successful ongoing and clinical pregnancy per single thawed oocyte is 7% in contrast to the equivalent rate when slow freezing is employed which is only 2.3% [62]. For that reason, women who want to cryopreserve would benefit from embarking on further ovarian stimulation protocols so as to ascertain a sufficient number of oocytes in order to improve their chances. It has been reported that, when the woman is >36 years old, more than 8 oocytes are required to be vitrified in order to improve the pregnancy rate [25].

There is no need for oocyte cryopreservation for social reasons to go hand in hand with a drop in the egg donation demand as they address different target groups that only overlap partly and hence they do not exclude one another. In fact, one could see egg donation as the last resort and a realistic option, following an unsuccessful use of cryopreserved oocytes for social reasons, but let us not forget that egg donation addresses and offers solutions for a variety of patients for a variety of reasons, that do not solely include social grounds. Although it initially appears that oocyte freezing may overshadow egg donation, we need to evaluate this on a long-term basis. An important factor to consider here is the age at which women opt for elective oocyte vitrification. From a biological and reproductive point of view, specialists would advise that optimum benefits could be enjoyed when oocyte vitrification is elected by a woman below the reproductive threshold/cut off age point—i.e., the age of 35 or even below the age of 30 [63]. Perhaps that will be the stronger and most common case in a few years with elective freezing becoming more well-known and available to the general female population. However, our clinical experience in 2017 supports that it is likely that women over the age of 35 and quite often closer to 40 will turn to elective freezing. This could be attributed to the fact that the reality of our true reproductive potential is not acknowledged by the general population. This fact is placing them within a non-functioning unrealistic timeframe, as they falsely identify the age of menopause as a possible age to turn to assisted reproduction.

### 3.3. The Link between Oocyte Vitrification for Social Reasons and Surrogacy

Another concern raised could be the attitude of women of reproductive age towards surrogacy following the thawing procedure of their cryopreserved oocytes. Will these women be tempted by the idea of surrogate motherhood? And if so, is this the beginning of a new tendency? Social egg freezing may enrich reproductive options and potential. When the time comes for these oocytes to be used, there is a good possibility for the maternal age to be advanced. As these women could probably be in their mid-40s, what could that mean and how will it be translated regarding the option of surrogacy? Could they consider surrogacy seriously as an option based on desire and not necessity. The option of surrogacy on the grounds of desire and not medical necessity could be linked to issues of varying nature and asymmetry as the driver behind such a decision. This rather rigid hypothesis may possibly reflect the thesis of a percentage of women that have opted for oocyte freezing on the grounds of a hard-driven career that perhaps does not allow ‘time off for motherhood’. Moreover, a pregnancy at the age of 40 is considered a condition of high risk, both for the mother and the fetus. Should we anticipate a rise in surrogacy following social egg freezing? This could be medically justified for certain cases but equally an option based on desire for some. This of course adds another level of complexity to the situation as surrogacy comes hand in hand with possible complications on various levels, from medical, to legal, to ethical depending on the case. However, to date surrogacy does not seem to be a popular alternative among women of reproductive age, as they would rather opt for uterus transplantation instead, with a percentage of 80% vs. 47% [64]. To further that point, it should be taken into account that in Sweden, where the data come from, surrogacy is not an available option due to Swedish legislation. As social egg freezing is a new trend, there is a strong need for more data to be collected about the long-term changes that it is about to induce in an effort to be well prepared.

### 3.4. Raising Awareness and Communicating Promotion of Oocyte Cryopreservation

Until recently, there has been little to offer to women who chose to postpone their motherhood, resulting in rising numbers of childless couples [65]. It is very important that women receive the correct information about oocyte cryopreservation and its success rates, and do not accept it as an ‘insurance’ as it may often be described [58]. Women typically go through the professional and reproductive ‘fertile’ period of their lives simultaneously. Proactive stance on motherhood regarding a woman excelling in her business domain while considering motherhood has lately gained the attention of large multinational companies resulting in the announcement of covering the costs of social egg freezing for their female employees. Women may feel pressure about childbearing especially when companies like Apple and Facebook offer to pay for social egg freezing treatments to their employees [66]. Followed by national campaigns, as in the case of the United States but also European countries like Switzerland [18], emphasis is focused on the promotion of social freezing through articles in the lay press, communicating the subject, followed by lectures addressed to lay people by centers providing services of oocyte freezing. Information is also widespread on the Internet, connecting oocyte cryopreservation with “freedom to choose how to spend one’s life, realising one’s full potential and gaining fertility freedom” [18].

At this point we should refer to the ‘power of the internet’ and social media. Even if elective freezing is considered to be a “special” category, there is a fine line between promoting knowledge and information in an appropriate and soundly controlled fashion and advertising a medical treatment. Admittedly, the internet is where the impressive majority ‘turns to’ in order to receive primary information. Consequently, one may rely on such information in order to make a choice on a medical treatment. There is no doubt that our era, is a time of abundant information readily available for all. Communication is simplified, anonymous, fast and easy. However, it should not be overlooked that all this information that is available on the internet and the social media could potentially be misleading. Blogs and chatrooms, when not properly supervised and controlled, could serve as facilitators of miscommunication. Medical advice should always be offered in a legal fashion, never jeopardizing the patients’ wellbeing.

In light of the controversial nature of social egg freezing, with competing perspectives and information available from a variety of sources, family physicians have a unique opportunity to assist women in accessing accurate and balanced information about their reproductive health. This information should be provided to all women who inquire about social egg freezing, regardless of sexual orientation, age, disability, health, relationship, or socioeconomic status. Family physicians should frame discussions about this practice within the broader context of reproductive health and family-planning to assist women in making informed choices [67].

However, public awareness should also be accurately positioned regarding the age limitations of egg freezing, assuring that the procedure is employed by those women who are most likely to benefit from it, that is to say women whose oocytes have not biologically substantially aged [68,69,70]. At the present level of efficacy of oocyte freezing, it is essential for women over 35 years of age to be made aware that their deposited eggs are not an insurance policy against age-related infertility [12]. It is essential to communicate that oocyte cryopreservation may serve as an informed subjective decision to possibly postpone motherhood for several more years. Considering the balance of benefits versus burdens and risks that oocyte cryopreservation incorporates at the present time is key.

It should be the ART world’s responsibility to ensure availability of extensive counselling and consent to be given first priority for women who wish to access this service. Our aim should include promoting long-term reproductive plans and providing a realistic assessment of the potential failures of oocyte freezing [70]. Counseling of all couples considering these procedures should include discussions of short- and long-term parenting and child rearing [15]. The increase in the proliferation of the information concerning the general subject matter of oocyte cryopreservation is noteworthy. Thus, it is indispensable to explore not only the medical and social advantages it entails, but also to review the implications resulting in substantial debates within the scientific community.

## 4. Societal, Psychological and Bioethical Concerns

### 4.1. How Women Perceive Social Freezing

Social oocyte freezing appears to be an insurance plan against age declining fertility; however, there are no guarantees. Prior to that, egg donation was an option to approach motherhood for women reaching the end of their reproductive span but lacking the genetic bond that remains desired.

A thought-provoking survey was carried out in Belgium regarding the intentions and attitudes of women of reproductive age regarding cryopreservation for non-medical reasons [71]. The study indicated that public awareness about oocyte cryopreservation effectively led to an increase of willingness to receive additional information on the subject for women of 38 years on average, potentially wishing to vitrify oocytes. According to the survey, 77.6% of the women had been previously aware of the technique and 31.5% of women would potentially cryopreserve their oocytes [71]. A smaller survey of 129 medical students in Singapore reported that 36.4% of respondents were familiar with the technique and 26.4% would consider it [72]. By the same token, reassurance about the possible risks to their future fertility related to the procedure, as well as the health safety of their children resulting from frozen eggs, appeared to enhance willingness to follow cryopreservation.

According to Stoop et al. (2011), single, non-cohabitating, or cohabitating women of higher educational status—mainly self-employed—were most probably prone to choose oocyte freezing [71]. The same cluster of women, possibly interested in social oocyte freezing, was also more open to donate oocytes. Similarly, regarding attitudes on potential egg freezing in Sweden, urban women of 30 to 39 years of age tend to be more positive towards various novel fertility treatment alternatives including oocyte cryopreservation [64].

In a more recent survey conducted in Belgium [17], they recorded the attitudes of women who aimed to cryopreserve their oocytes at the time of oocyte retrieval/freezing and at an interval of 12–45 months later. They also recorded the attitude of women who did not want to freeze towards elective oocyte freezing. Both study groups regarded the most appealing alternative choice to be IUI with donor sperm and second most popular option, adoption. Of the number of women who cryopreserved their oocytes, only half of them actually believed that they would use them in the future. In total, 30% of oocyte bankers three years following oocyte retrieval still believed in the use of those oocytes in order to become pregnant. Neither of the oocyte bankers stated any regrets regarding their decision. The only thing they would have reconsidered is the age they were when they opted to pursue this option [17]. In a more recent cohort study in Germany a total of 643 participated in order to investigate relations between attitudes toward social oocyte freezing and different socio-cultural background. They reported a clear link between attitudes towards social oocyte freezing and socio-cultural background, gender, age, fertility problems, and attitudes to fertility [73].

### 4.2. Reproductive Autonomy and Emerging Conflicting Bioethical Issues

Women, biologically have a more limited reproductive lifespan compared to men, dealing with further difficulties in conceiving as age progresses. Gosden et al. [74] referred to this situation as ‘biological inequity’. This is the very reason why women in their late 30s and early 40s are overrepresented in fertility clinics [71]. Regarding the element of social and personal sense of stigmatization in connection to fertility challenges, Becker [75] described how inability to have children disrupts women’s gender identities. Hence, women may view infertility as a greater tragedy compared to their male partners [76]. They report feeling that the inability to bear children has “stigmatized” and “spoiled their identities” as women, thus taking on more responsibility in undergoing fertility treatments.

By the same token, when faced with fertility issues, women often tend to use words such as “failure” and “broken” when describing their bodies, compared to men who refer to their infertility as “emasculating” [77]. Oocyte freezing, as a means or preservation of fertility, may be well serving as the link which proactively or actively connects women back to reproduction. This link may function as an ‘equalizer’, in comparison to men, as far as reproductive lifespan is concerned.

This effectively leads to the search for “reproductive autonomy”; namely, the freedom to decide on whether, with whom and when to have children. Reproductive autonomy is an important value in modern western societies, as is the protected right to “establish a family” based on the European Convention for Human Rights [61].

An important ethical issue refers to the well-being of the child, product of social egg freezing. The medical risks to the mother and child have been thoroughly discussed and are of great significance; however, their thorough evaluation will be possible following analysis and understanding of adequate data that is still recorded. The fundamental ethical issue is whether the interests of women and children are served by the use of this technology. These interests can be served when the desire for a child and the ultimate bearing and rearing of a child contribute to mutual wellbeing (Ethics Committee of the American Society of Reproductive Medicine, 2013).

In Switzerland, the law proclaims that the well-being of the future child is the first and supreme principle. Therefore, elevated risk of “potential medical complications for the future should be avoided” [18]. Parental old age which could be going hand in hand with oocyte freezing for social reasons is a serious matter to consider. One could argue that it should be vital for the child’s well-being to have young and healthy parents. Late pregnancies raise serious concerns about maternal risks of pregnancy at a higher age and about the negative psychosocial consequences for the child [78].

To build on that concept it should be considered that maternal older age and pregnancy, may translate to difficulties in organising career and raising children which become even greater, especially when older couples are involved or when contemplating single parenthood. Further to that, delayed motherhood renders the state of health of grandparents perhaps incompatible. It should also not be neglected that children of older parents are reposted to run the risk of psychological problems resulting from “shame” as their parents could often be mistaken as being their grandparents [79].

One cannot help but ponder on the burden of responsibility of the modern society on the escalation of such complex matters. Modern societies are organised in ways that simultaneously challenge and enable women. This fact refers to achieving the golden triptych standard between (1) following a promising professional career; (2) selecting the partner with whom they feel secure and comfortable to start their families with; and at the same time (3) abide by the somewhat strict but realistic biological reproductive lifespan so as to enter motherhood in a balanced and productive fashion.

The reality of limitations of the human life is difficult to accept; older age and the natural reproductive consequences it entails may be put aside by elective oocyte vitrification. Martin et al., (2010) argues that “medical advancements could split the woman’s body in two; her younger self, the egg donor, and her older self, the recipient” [76].

### 4.3. Analyzing the Social, Educational, and Psychological Profiles of Women

Martin (2010) proposes an ontological categorisation of women “anticipating infertility” rather than being exposed to it. This serves as a sociological descriptor of the phenomenon which we attempt to explore [76]. Elective oocyte vitrification addresses mainly women who seek a stress-relief from the age-related social pressure to enter motherhood. For these women issues such as professional and financial stability and the existence of a supportive, long-term relationship remain unpredictable and volatile. Similar to sperm banking and invoking greater gender equality, egg freezing may enable women to preserve and store their oocytes for future use. This in turn enables them to address the basic sociocultural anxieties involving aging, illness and reproduction limitation. Increased control gain over women’s own reproductive future, possibly enables them to achieve several goals before willingly entering motherhood. These commonly refer to more time to find a suitable partner, to complete education and achieve financial and psychological stability [61].

The factor of age-related decline in fertility appears to be the main reason the majority of women’s attention considering self-donation of oocytes is captured. The sense of regained control over their reproductive future is the main asset this method proclaims. It is also what has reasonably initiated the widespread debate regarding the social and psychological repercussions following egg freezing [58,68,79].

According to Lockwood (2011), an “increasingly numerically and economically group of young adults” are steadily moving towards an “ambivalent” attitude to parenthood/childlessness, described as “perpetual postponing”, concerning both men and women [58]. This group, especially if they have received a higher level of education and have secured good professional future prospects, remain unprepared to fulfil the parental role. Especially when considering the narrow time space of opportunity which exists between the realization of educational and professional goals, and the beginning of a family, voluntary or involuntary childlessness becomes commonplace. The women in this group, as Lockwood explains, “maintain a latent desire for motherhood but do not act upon it until it could be too late in biological time”. Thus, the rise in constant postponers has reasonably increased dependence on assisted conception.

Another compelling social factor contributing to choosing to oocyte cryopreservation is the lack of a long-term relationship. Failing to find a partner wishing to head towards parenthood with, or general commitment issues, affects the time of the realization of a pregnancy.

Additionally, as Lockwood (2011) explains, in the most recent years, there has been an emergence of what the researcher calls a “flight from parenthood” rather than the “delay and catch up”. The chance of childlessness for women at the age of 45 having received a higher educational level is significantly higher than for women without qualifications. This fact points to the low pregnancy success rates for women over 40 using their own eggs in ART. It becomes crucial, therefore, as Lockwood (2011) proposes, that fertility specialists responsibly inform their patients that a woman over 40 is more likely to achieve a healthy pregnancy using embryos or oocytes originating from her mid-30s than trying IVF or ICSI with fresh over-40-year-old oocytes [58].

The factor of desire to enter parenthood plays a significant role. Younger women with a largely unfulfilled desire for children are more open to the idea of oocyte cryopreservation, contrary to women not having reached a firm decision on the matter of desiring a child at any point of their lives. This fact highlights the important factor of willingly wishing to become a mother, given the right circumstances [71]. In an analogous way, potential freezers appear to almost having a decade before confronted with age-related fertility decline while planning to start their family at a later stage in life, which could possibly be explained by educational, professional, and financial aspects.

At this point it is important to describe a ‘best-case scenario’. Step one, realization: a woman in her late 20s/early 30s realizes that her desire to become a mother does not coincide with her social and financial situation. Step two, acknowledgement: it is essential that she acknowledges the fact that by the time her aspirations and relational status have reached a point she may be comfortable with, her oocytes will unfortunately have aged, leading to considerably lower implantation rates and a higher risk of chromosomal abnormalities. Step three, proactive action: she would be inclined to act proactively and cryopreserve young oocytes in order to achieve a pregnancy at a later point in life [68].

## 5. Conclusions

Oocyte cryopreservation on medical grounds is indisputably an advantageous linear solution and well-accepted socially [80]. From a medical, psychological and bioethical stand point, oocyte freezing for nonmedical reasons raises controversy within the scientific community due to the multifaceted underlying reasons evoking it. Societal changes and medical developments in recent years have given rise to the augmenting design of services addressed to women opting for ‘social egg freezing’. The mere observation that clusters of women tend to postpone motherhood and consider going through ART to cryopreserve their oocytes, renders the current review of medical, social and psychological aspects of this group essential and timely.

The aspects analyzed move on the axis of the general reproductive health and its implications. The relevant subsections refer to how the ART set up and practice may be affected. From the application of invasive techniques such as ICSI and PGD/PGS, to the effect of the extra manipulation on the embryos’ identity on an epigenetic level. Considerations on obstetrics as well as the fetal and perinatal matters are analyzed, focusing on the advantages and risks that could be involved from a more horizontal application of the service and the options enabled for the women opting for it. Finally, the psychological profile and data on social freezing are presented, while the social and bioethical issues raised are inclusively deliberated.

Although vitrification is an established technique in the IVF laboratories with comparable results to fresh cycles there is still room from large scale trials to clarify the possibly “cloaked” negative impact on oocyte physiology [55]. This new trend will lead to a horizontal application of ICSI and multiple vitrifications, focusing our attention on epigenetic impacts on the future offspring. Further to that, this trend is related to women of advanced maternal age at the anticipated time of employment of the cryopreserved oocytes and subsequently revealing a link to the possibility of increased risk of gestational diabetes, preeclampsia, preterm delivery, and low birth weight, along with an increasing number of elective cesarean sections performed. On the other hand, interestingly, the existing perinatal data do not show any added complications and serve to support safe application of oocyte vitrification [47]. Considering the possible effect of this new trend on the egg donation programs, the literature failed to present a clear link strengthening the scenario that oocyte donation demand remains unaffected as a separate option representing the last resort on the infertile women’s journey. Association of the trend with an increased option of surrogacy may be a valid hypothesis. On the less clinical, but extremely important note, of the socio-psychological and bioethical scope, a wide range of publications support that women of a high educational and socio-economical profile tend to present with a more positive attitude towards oocyte freezing for social reasons [64]; exploring the strengths, weaknesses, and overall boundaries of their reproductive autonomy while the socio-cultural surroundings remain the ultimate drivers fueling reproductive decisions. In conclusion, this thorough literature review on this multifactorial phenomenon of social oocyte freezing emphasizes both positive and negative respects and highlights the obligation of the ART scientific community in raising awareness and providing information towards informed consent regarding oocyte cryopreservation.

This literature review collectively presents all aspects related to the practise of elective oocyte cryopreservation as well as the implications involved on all levels approached. How we expect elective oocyte cryopreservation to change the scenery as we know it is to be seen. Taking into consideration the embryological, genetic, obstetric and perinatal points of view to the psychological and bioethical standpoint is crucial. The limitations and considerations on this contemporary growing trend are thoroughly contemplated, as well as the benefits and the changes currently experienced and expected in the future.

## Supplementary Materials

The following are available online at http://www.mdpi.com/1010-660X/54/5/76/s1.

## Abbreviations

COH	controlled ovarian stimulation
POF	premature ovarian failure
IVF	in vitro fertilization
ART	artificial reproductive technology
CPA	cryoprotective agents
ICSI	intra cytoplasmic sperm injection
PGD	preimplantation genetic diagnosis
PGS	preimplantation genetic screening
UKOSS	UK Obstetric Surveillance System
