# Supplementary material for: Postponing Pregnancy Through Oocyte Cryopreservation for Social Reasons: Considerations Regarding Clinical Practice and the Socio-Psychological and Bioethical Issues Involved
Source: Medicina (Kaunas). 2018 Oct 25;54(5):76. doi: 10.3390/medicina54050076 (PMC6262467; doi:10.3390/medicina54050076)
Supplement: Supplementary file 1 [file medicina-54-00076-s001.pdf]

Supplementary Table 1

Search Strategy for Pubmed/Medline as performed on the 23rd of June 2018.

1. Freezing OR cryopreservation OR vitrification
2. #1 AND oocyte
3. #1 AND social
4. #2 AND #3
5. #2 AND bioethics
6. #2 AND psychosocial
7. #4 OR #5 OR #6
